# A Nonlinear Transfer Operator Theorem

**DOI:** 10.1007/s10955-016-1646-1

**Published:** 2016-11-09

**Authors:** Mark Pollicott

**Affiliations:** Mathematics Institute, University of Warwick, Coventry, CV4 7AL UK

**Keywords:** Ruelle operator theorem, Transfer operator, Thermodynamic formalism

## Abstract

In recent papers, Kenyon et al. (Ergod Theory Dyn Syst 32:1567–1584 2012), and Fan et al. (C R Math Acad Sci Paris 349:961–964 2011, Adv Math 295:271–333 2016) introduced a form of non-linear thermodynamic formalism based on solutions to a non-linear equation using matrices. In this note we consider the more general setting of Hölder continuous functions.

## Introduction

We first recall a classical result for matrices dating back to work of Perron (1907) and Frobenius (1912) (cf. [5], p. 53). A $k \times k$ matrix *A* is called *non-negative* if all the entries are non-negative real numbers and *aperiodic* if there exists $n>0$ such that all entries of the *n*th power $A^n$ are strictly positive.

### Theorem 1.1

(Perron–Frobenius Theorem) Let *A* be a non-negative aperiodic $k \times k$-matrix. There exists a unique positive maximal eigenvalue $\lambda > 0$ and a unique positive eigenvector $\underline{v}$ such that $A\underline{v} = \lambda \underline{v}$.

We next recall a generalisation of the Perron–Frobenius Theorem to Banach spaces of functions. Let $\sigma : \Sigma \rightarrow \Sigma$ be a one-sided mixing subshift of finite type with alphabet $F = \{1, \cdots , k\}$ (i.e., there exists an aperiodic $k \times k$ matrix *B* with 0–1 entries such that $\Sigma = \{ x = (x_n)_{n=0}^\infty \in \prod _{n=0}^\infty F \hbox { : } x_n \in F, B(x_n, x_{n+1}) = 1, \forall n \ge 0\}$ and $(\sigma x)_n = x_{n+1}$. Given $0< \theta < 1$, let $\mathcal F_\theta$ be the space of functions $f: \Sigma \rightarrow {\mathbb {R}}$ for which the semi-norm

$$\begin{aligned} \Vert f\Vert _\theta = \sup _{n \ge 0} \frac{\hbox {var}_n(f)}{\theta ^n} < +\infty \end{aligned}$$

is finite, where $\hbox {var}_n(f) = \sup \{|f(x) - f(x^{\prime })| \hbox { : } x_i = x^{\prime }_i \hbox { for } i =0, \cdots , n-1\}$. In particular, $\mathcal F_\theta$ is a Banach space with respect to the norm $\Vert f\Vert = \Vert f\Vert _\theta + \Vert f\Vert _\infty$.

### Definition 1.2

Let $\phi \in \mathcal F_\theta$. We can define a transfer operator $\mathcal L_\phi : \mathcal F_\theta \rightarrow \mathcal F_\theta$ by

$$\begin{aligned} \mathcal L_\phi \psi (x) = \sum _{\sigma y = x} e^{\phi (y)} \psi (y), \end{aligned}$$

where $$\psi \in \mathcal F_\theta $$ and $$x\in \Sigma $$.

The following result of Ruelle is a cornerstone of the classical theory of thermodynamic formalism (cf.[10]).

### Theorem 1.3

(Ruelle–Perron–Frobenius Theorem) Let $\phi \in \mathcal F_\theta$.

1. There exists $\lambda = \lambda _\phi > 0$ and $\varphi = \varphi _\phi \in \mathcal F_\theta$ with $\psi > 0$ such that $\mathcal L_\phi \varphi = \lambda \varphi$;
2. any $\psi ^{\prime } \in \mathcal F_\theta$ with $\mathcal L_\phi \psi ^{\prime } = \lambda \psi ^{\prime }$ is necessarily a multiple of $\psi$; and
3. the dependences $\mathcal F_\theta \ni \phi \mapsto \lambda _\phi \in \mathbb R^+$ and $\mathcal F_\theta \ni \phi \mapsto \varphi _\phi \in \mathcal F_\theta$ are analytic.

It is also known that, aside from the maximal eigenvalue $\lambda$, the rest of the spectrum of $\mathcal L_\phi : \mathcal F_\theta \rightarrow \mathcal F_\theta$ is contained in $\{z \in \mathbb C \hbox { : } |z| < \lambda _\phi \}$. In particular, part 3 of Theorem 1.3 then follows from part 1 by standard perturbation theory.

The value $P(\phi ):= \log \lambda _\phi$ is called the *pressure* of the function $\phi \in \mathcal F_\theta$ [10]. In the case that $\phi (x) = \phi (x_0,x_1)$ depends only on the first two terms in $x = (x_n)_{n=0}^\infty$ then Theorem 1.3 reduces to Theorem 1.1, by taking $A(i,j) = \exp {\phi (i,j)}$. In this case, $\psi (x) = \psi (x_0)$ depends only on the first term and we can set $\underline{v} = (\psi (1), \ldots , \psi (k))$.

Recently, several authors introduced a particular non-linear version of Theorem 1.1 for matrices which is useful in the study of the dimension of certain sets in the theory of non standard ergodic averages (see section 2).

### Theorem 1.4

(Kenyon–Peres–Solomyak, Fan–Schmeling–Wu) Let *B* be a non-negative irreducible $k \times k$ matrix. There exists a unique positive vector $\underline{v}$ such that $B\underline{v} = \underline{v}^2$, where the entries of $\underline{v}^2$ are the square of those of $\underline{v}$, i.e., ${(\underline{v})_i}^2 = v_i$ for $i=1, \cdots , k$.

In the special case that *A* has entries which are natural numbers, this appears as Lemma 1.2 in [6]. A version of this for more general positive matrices appears as 4.1 in [2] (cf. also [3]) under very modest assumptions on the matrix. Other types of non-linear Perron–Frobenius Theorem appear in [7] and [8].

The following is our main result, which can be viewed either as a non-linear version of Theorem 1.3, or a generalisation of the Theorem 1.4 (at least for aperiodic matrices) from matrices to functions.

### Theorem 1.5

(Main Theorem) Let $\phi \in \mathcal F_\theta$.

1. There exists $\psi = \psi _\phi \in \mathcal F_\theta$ with $\psi > 0$ such that $\mathcal L_\phi \psi = \psi ^2$;
2. for any $\psi ^{\prime } \in \mathcal F_\theta$ with $\mathcal L_\phi \psi ' = {\psi ^{\prime }}^2$ and $\psi ' > 0$ then $\psi ' = \psi$; and
3. the dependence $\mathcal F_\theta \ni \phi \mapsto \psi _\phi \in \mathcal F_\theta$ is analytic providing $\psi _\phi$ is sufficiently close to the constant function $\lambda _\phi \mathbf 1$ in norm.

The result easily generalises to $\mathcal L_\phi \psi = \psi ^q$, for any natural number $q \ge 2$. We consider only the case $q=2$ to avoid introducing additional notation.

In the particular case that the function $\phi (x) = \phi (x_0,x_{1})$ depends on only finitely many coordinates then Theorem 1.4 can be recovered as a corollary to Theorem 1.5.

### Example 1.6

**Fig. 1:**
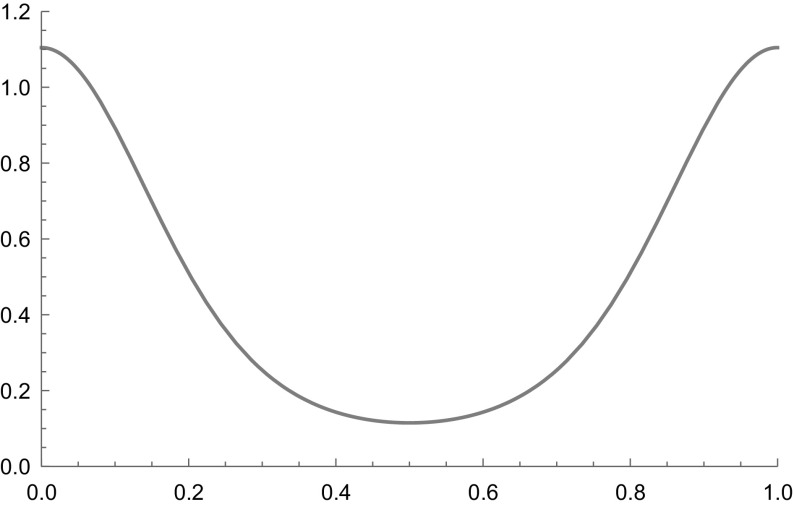
A plot of $\psi _\phi \left( x\right)$ using the dyadic expansion $0 \le \sum _{n=0}^\infty \frac{(x_n-1)}{2^{n+1}}\le 1$ on the unit interval to represent $x\in \Sigma _2$

Let $\Sigma _2 = \{1,2\}^{\mathbb Z_+}$ correspond to a full shift with $F = \{1,2\}$. We can define a function $\phi : \Sigma _ 2\rightarrow \mathbb R$ by

$$\begin{aligned} \phi (x) = - 4 \sin \left( 2 \pi \sum _{n=0}^\infty \frac{(x_n-1)}{2^{n+1}}\right) \end{aligned}$$

and observe that $\phi \in \mathcal F_\theta$ for any $1/2< \theta < 1$. By Theorem 1.5 (with the choice $q=2$) there is a function $\psi$ such that $\mathcal L_\phi \psi = \psi ^2$. In Fig. 1 we plot a realisation of $\psi$ using the dyadic expansion on the unit interval.

### Remark 1.7

If $\phi \in \mathcal F_\theta$ then, as usual, by replacing $\phi$ by $\phi _1 = \phi + \log \psi _\phi - \log \psi _\phi \circ \sigma \in \mathcal F_\theta$, where $\psi$ is the positive eigenfunction in Theorem 1.3, we can assume without loss of generality that $\psi _{\phi _1}(x) = \mathbf 1$ is the constant function taking the value 1, i.e., $\mathcal L_{\phi _1} \mathbf 1 = \lambda \mathbf 1$, where $\lambda = \lambda _{\phi } = \lambda _{\phi _1}$. In particular, for such special normalized functions $\phi _1$ the function $\psi$ Theorem 1.5 can easily be identified as $\psi = \psi _{\phi _1} = \lambda \mathbf 1$, then we see that $\mathcal L_{\phi _1} \psi = \psi ^2$. Furthermore, the hypothesis for analyticity in part 3 of Theorem 1.5 automatically holds.

I am grateful to the referees and the editors for their patience and help with this short note.

## Background to Theorem 1.4

Although our main result (Theorem 1.5) is of independent interest, for the reader’s benefit we will now give a brief description of the original application of its precursor (Theorem 1.4) which provided the motivation for its introduction.

Following [6] and [2, 3] given a probability measure $\mu$ on $\Sigma$ we can define a so-called multiplicative measure $\nu$ on $\Sigma = \{1, \ldots , k\}^{\mathbb Z^+}$, say, by writing $\Sigma = \prod _{j \hbox { odd}}\Sigma _j$ where $\Sigma _j = \{1, \cdots , k\}^{\Lambda _{j}}$ and $\Lambda _{j}$ = $\{j 2^n \hbox { : } n \ge 0\}$, for $j = 1,3, 5, \cdots$, which form a natural partition of $\mathbb N$ by ${\mathbb {N}} = \cup _{j \hbox { odd}}\Lambda _{j}$. We can then define $\nu = \prod _{j} \mu$, in a natural sense. In [6] and [3] the authors consider the measure $\mu$ to be a (generalised) Markov measure defined in terms of the entries in the vector $\underline{v}$ in Theorem 1.4. The measure $\nu$ will typically not be $\sigma$-invariant but is still useful in studying the Hausdorff dimension of certain sets.

We can define the pointwise dimension of $\nu$ by

$$\begin{aligned} \text {dim}_H (\nu ) = - \lim _{n\rightarrow +\infty } \frac{1}{n}\log \nu \left( [x_0, x_1, \cdots , x_{n-1}]\right) \hbox { for a.e.}(\nu ) x = (x_n) \in \Sigma \end{aligned}$$

where $[x_0, x_1, \cdots , x_{n-1}] =\{y = (y_n)\in \Sigma \hbox { : } x_i = y_i, 0 \le i \le n-1\}$ is a cylinder set. Finally, by Proposition 2.3 of [6] we have that for any $\sigma$-ergodic measure $\nu$ the pointwise dimension is constant and takes the explicit value

$$\begin{aligned} \text {dim}_H (\nu ) = \sum _{n=1}^\infty \frac{1}{2^{n+1}} H_\mu \left( \vee _{i=0}^{n-1} \sigma ^{-i}\alpha \right) \end{aligned}$$

where $\alpha = \{[1], \ldots , [k]\}$ is the standard partition into cylinders of length one; $\vee _{i=0}^{n-1} \sigma ^{-i}\alpha$ is the usual refinement to a partition by cylinders of length *n*; and $H_\mu (\cdot )$ is the entropy for partitions [11].

The pointwise dimension is particularly useful in estimating the Hausdorff Dimension of sets (especially lower bounds via the usual mass distribution principle cf. [1], §4.2) associated to multiple ergodic theorems, as the following example illustrates.

### Example 2.1

(Golden Mean Example [4, 6]) Fan–Liao–Ma and Kenyon–Peres–Solomyak considered the golden mean example:

$$\begin{aligned} X = \left\{ (x_n) \in \{0,1\}^{\mathbb N} \hbox { : } x_n x_{2n} = 0, \forall n \ge 1\right\} , \end{aligned}$$

with the usual metric

$$\begin{aligned} d_\theta (x,x^{\prime }) = {\left\{ \begin{array}{ll}\theta ^{N(x,x^{\prime })} &{} \hbox { if } x \ne x^{\prime } \\ 0 &{} \hbox { if } x = x^{\prime }. \end{array}\right. } \end{aligned}$$

where $N(x,x^{\prime }) = \sup \{n \ge 0 \hbox { : } x_i = x^{\prime }_i \hbox { for } 0 \le i \le n\}$ (and $N(x,x^{\prime })=0$ if $x_0 \ne x^{\prime }_0$).

In this case one can consider the matrix $$B = \left( \begin{matrix} 1 &{} 1 \\ 1 &{} 0 \end{matrix} \right) $$ and the solution to $B \underline{v} = \underline{v}^2$, i.e., $$v = \left( \begin{matrix}v_1 \\ v_2 \end{matrix} \right) $$ satisfies $v_1^2 = v_1 + v_2$ and $v_2^2 = v_1$. We then have that $\mu$ is a Markov measure for $$P= \left( \begin{matrix}p &{}\quad 1-p \\ 1 &{} 0 \end{matrix} \right) $$, where $p^3 = (1-p)^2$, and finally $\dim _H(X) = - \log _2 p = 0 \cdot 81137 \ldots$ which is strictly less than the Minkowski dimension $dim_M(X) = 0 \cdot 82429 \ldots$ [4, 6].

## Proof of Theorem 1.5

The proof of the existence of the fixed point is the more interesting part of the problem. The uniqueness and analyticity are then relatively easy to establish.

### Existence of the Fixed Point

The existence can be shown by looking for a fixed point of a suitable map in the space

$$\begin{aligned} \Lambda _c : = \left\{ u: \Sigma \rightarrow [0,1] \hbox { : } u(x) \le u(x^{\prime }) e^{cd_\theta (x,x^{\prime })} \hbox { for those } x,x^{\prime } \in \Sigma \hbox { with } x_0 = x^{\prime }_0 \right\} \end{aligned}$$

where $c > 0$ and $d_\theta (\cdot , \cdot )$ is as defined in Example 2.1. We first note that $\Lambda _c \subset \mathcal F_\theta$ since for $u\in \Lambda _c$ and $x,x^{\prime }\in \Sigma$ we can bound $u(x) - u(x^{\prime }) \le \Vert u\Vert _\infty \left( e^{c d_\theta (x,x^{\prime })} -1\right) \le C d_\theta (x,x^{\prime })$ for sufficiently large $C>0$ and then interchanging *x* and $x^{\prime }$ gives that $\Vert u\Vert _\theta \le C$ (cf. [9], p. 22).

We can now introduce a family of non-linear operators defined as follows:

#### Definition 3.1

For each $n \ge 1$ we can associate to $u \in \Lambda _c$ a new function $\mathcal N_n(u): \Sigma \rightarrow {\mathbb {R}}$ defined by

$$\begin{aligned} \mathcal N_n(u)(x) = \left( \frac{\mathcal L_\phi \left( u + \frac{1}{n} \mathbf 1 \right) (x) }{\Vert \mathcal L_\phi \left( u + \frac{1}{n} \mathbf 1 \right) \Vert _\infty }\right) ^\frac{1}{2} \end{aligned}$$

where $\frac{1}{n} \mathbf 1$ represents the function taking the constant value $\frac{1}{n}$.

#### Lemma 3.2

We have that $\mathcal L_\phi (\Lambda _c) \subset \Lambda _{c^{\prime }}$ for $c^{\prime } = (c + \Vert \phi \Vert _\theta )\theta$.

#### Proof

Let $x,x^{\prime } \in \Sigma$ with $x_0 = x^{\prime }_0$. Assume that $d_\theta (x,x^{\prime }) = \theta ^N$, for some $N \ge 0$, then $x_i = x^{\prime }_i$ for $0 \le i \le N$ and $x_{N+1} \ne x^{\prime }_{N+1}$. If $y \in \sigma ^{-1}x$ then we denote by $y^{\prime } \in \sigma ^{-1}x^{\prime }$ the corresponding sequence for which $y_0=y^{\prime }_0$, and thus we have that $d_\theta (y,y^{\prime }) = \theta ^{N+1}$. Let $u \in \Lambda _c$ then we have that

$$\begin{aligned} \begin{array}{ll} \mathcal L_\phi u (x) &{}= \sum _{\sigma y = x} e^{\phi (y)} u(y) \\ &{}\le \sum _{\sigma y = x} e^{\phi (y^{\prime }) + \Vert \phi \Vert _\theta \theta ^{N+1}} \left( u(y^{\prime }) e^{c\theta ^{N+1}} \right) \\ &{}\le e^{(c + \Vert \phi \Vert _\theta ) \theta d_\theta (x,x^{\prime })}\sum _{\sigma y^{\prime } = x^{\prime }} e^{\phi (y^{\prime })} u(y^{\prime }) \\ &{}= e^{(c + \Vert \phi \Vert _\theta )\theta d_\theta (x,x^{\prime })} \mathcal L_\phi u (x^{\prime }) \\ \end{array} \end{aligned}$$

where we have used that $d_\theta (y,y^{\prime }) = \theta ^{N+1}$ and then since $u \in \Lambda _c$ we have that $u(y) \le u(y^{\prime }) e^{c \theta ^{N+1}}$. In particular, we have that $\mathcal L_\phi u (x) \le e^{c^{\prime } d_\theta (x,x^{\prime })} \mathcal L_\phi u (x^{\prime })$, i.e., $\mathcal L_\phi u \in \Lambda _{c^{\prime }}$. $\square$

#### Remark 3.3

By definition of $\Lambda _c$, we see that if $c^{\prime } < c$ then $\Lambda _{c^{\prime }} \subset \Lambda _{c}$ thus, providing *c* is sufficiently large, Lemma 3.2 gives $\mathcal L_\phi (\Lambda _c) \subset \Lambda _c$.

We can use the above lemma to deduce the following.

#### Lemma 3.4

For $c>0$ sufficiently large we have that $\mathcal N_\phi (\Lambda _{c}) \subset \Lambda _c$.

#### Proof

Let $u \in \Lambda _c$. For each $n \ge 1$ the constant function $\frac{1}{n} \mathbf 1 \in \Lambda _c$ and so by applying Lemma 3.2 to the new function $u + \frac{1}{n} \mathbf 1$ we see that

3.1 $$\begin{aligned} \mathcal L_\phi \left( u + \frac{1}{n} \mathbf 1\right) (x) \le e^{c^{\prime } d_\theta (x,x^{\prime })} \mathcal L_\phi \left( u + \frac{1}{n} \mathbf 1\right) (x^{\prime }), \end{aligned}$$

for all $x,x^{\prime } \in \Sigma$ with $x_0 =x^{\prime }_0$. Dividing both sides of (3.1) by $\Vert \mathcal L \left( u + \frac{1}{n} \mathbf 1\right) \Vert _\infty \ > 0$ we have that

3.2 $$\begin{aligned} \frac{\mathcal L_\phi \left( u + \frac{1}{n}\mathbf 1 \right) (x)}{\Vert \mathcal L \left( u + \frac{1}{n} \mathbf 1\right) \Vert _\infty } \le e^{c^{\prime } d_\theta (x,x^{\prime })} \frac{\mathcal L_\phi \left( u + \frac{1}{n} \mathbf 1\right) (x^{\prime })}{\Vert \mathcal L \left( u + \frac{1}{n} \mathbf 1 \right) \Vert _\infty }. \end{aligned}$$

Finally, since the values taken by both sides of (3.2) lie in the unit interval, taking square roots preserves this property with $c^{\prime }$ replaced by $c^{\prime }/2$, i.e.,

$$\begin{aligned} \begin{array}{ll} {\mathcal N}_n(u)(x) = \left( \frac{\mathcal L_\phi \left( u + \frac{1}{n}\mathbf 1\right) (x)}{\Vert \mathcal L_\phi \left( u + \frac{1}{n}\mathbf 1\right) \Vert _\infty } \right) ^\frac{1}{2} &{}\le e^{(c^{\prime }/2)d_\theta (x,x^{\prime })} \left( \frac{\mathcal L_\phi \left( u + \frac{1}{n}\mathbf 1 \right) (x^{\prime })}{\Vert \mathcal L_\phi \left( u + \frac{1}{n}\mathbf 1\right) \Vert _\infty }\right) ^\frac{1}{2}\\ &{}= e^{(c^{\prime } /2)d_\theta (x,x^{\prime })} {\mathcal N}_n(u)(x') \end{array} \end{aligned}$$

i.e., $N_n\left( u\right) \in \Lambda _{c^{\prime }/2}$. Providing *c* is sufficiently large that $c > c^{\prime }/2 = (c+ \Vert \phi \Vert _\theta )\theta /2$ we have that $\Lambda _{c^{\prime }/2} \subset \Lambda _c$ and the result follows. $\square$

This now brings us to the existence of the fixed point for each of the operators $\mathcal N_n: \Lambda _c \rightarrow \Lambda _c$.

#### Lemma 3.5

For each $n \ge 1$, there exists a non-trivial fixed point $\psi _n \in \Lambda _c$ such that $\mathcal N_n(\psi _n) = \psi _n$.

#### Proof

By the Arzela–Ascoli Theorem the space $\Lambda _c$ is compact with respect to the norm $\Vert \cdot \Vert _\infty$. For each $n \ge 1$ and $c>0$ sufficiently large the map $\mathcal N_n: \Lambda _c \rightarrow \Lambda _c$ is a continuous map on a compact convex subspace of $C^0(\Sigma )$ and we can apply the Schauder fixed point theorem to deduce that there is a fixed point $\psi _n \in \Lambda _c$ for $\mathcal N_n$. To see that $\psi _n$ is not identically zero we need only observe that by the definition of $\mathcal N_n$ there exists $x^{(n)}\in \Sigma$ with $\mathcal N_n(\psi _n)(x^{(n)}) =1$ and by construction $\psi _n(x^{(n)}) = \mathcal N_n(\psi _n)(x^{(n)}) =1$. This completes the proof. $\square$

We can now use the compactness of $\Lambda _c$ with respect to $\Vert \cdot \Vert _\infty$ to deduce that $(\psi _n)_{n=1}^\infty$ has a $\Vert \cdot \Vert _\infty$-convergent subsequence. We denote the limit point by $\psi _0 \in \Lambda$ and observe that we have that $\mathcal L_\phi (\psi _0) = \lambda \psi _0^2$, where $\lambda = \Vert \mathcal L_\phi (\psi _0)\Vert _\infty$. Moreover, since we observed that by construction that $\Vert \psi _n\Vert _\infty =1$, for each $n \ge 1$, we can deduce that $\Vert \psi _0\Vert _\infty =1$ and thus, in particular, $\psi _0$ is non-zero. If we replace $\psi _0$ by $\psi = \lambda \psi _0$ then we finally get $\mathcal L_\phi (\psi ) = \psi ^2$, as required.

To see that $\psi >0$, assume for a contradiction that there exists $x_0 \in \Sigma$ such that $\psi (x_0) =0$. Since $\psi \ge 0$ we see from the identity $\mathcal L_\phi (\psi )(x_0) = \psi ^2(x_0) = 0$, which implies that $\psi (y)=0$ whenever $\sigma y = x_0$. Proceeding iteratively, we have that $\psi$ vanishes on the set $\cup _{n=0}^\infty \sigma ^{-n} x_0$, which is dense by the mixing hypothesis on $\sigma : \Sigma \rightarrow \Sigma$ (corresponding to the aperiodicity assumption on *A*). However, this contradicts that $\psi \ne 0$.

### Uniqueness of the Positive Fixed Point

Assume for a contradiction that we had a second distinct non-trivial positive fixed point, i.e., $\mathcal L_\phi (\psi ^{\prime }) = {\psi '}^2$ with $\psi ^{\prime } > 0$ and $\psi \ne \psi ^{\prime }$. We can then associate $\xi := \inf \{t> 0 \hbox { : } t\psi - \psi ^{\prime } \ge 0 \}$ and thus, in particular, $\xi \psi \ge \psi ^{\prime }$. Observe that $\mathcal L_\phi (\xi \psi - \psi ^{\prime }) = \xi \psi ^2 - {\psi '}^2 \ge 0$, since $\mathcal L_\phi$ preserves positive functions. Since $\xi \psi ^2 - {\psi '}^2 = ( \sqrt{\xi } \psi + \psi ^{\prime })( \sqrt{\xi } \psi - \psi ^{\prime }) \ge 0$ we deduce that $\sqrt{\xi } \psi - \psi ^{\prime } \ge 0$. In particular, this implies that $\xi \le 1$, otherwise it contradicts the original definition of $\xi$.

Interchanging the roles of $\psi$ and $\psi ^{\prime }$ we can define $\xi ^{\prime } := \inf \{t> 0 \hbox { : } t\psi ^{\prime } - \psi \ge 0 \}$ and and thus, in particular, $\xi ^{\prime } \psi ^{\prime } \ge \psi \ge 0$. A similar argument to the above shows that $\xi ^{\prime } \le 1$. However, since we can then write $(\xi ^{\prime } \xi ) \psi ^{\prime } \ge \xi \psi \ge \psi ^{\prime }$ this implies that $\xi = \xi ^{\prime } = 1$.

For the definition of $\xi$ we can choose $x_0$ with $\psi (x_0) = \psi ^{\prime } (x_0)$. We can then write $\mathcal L_\phi ( \psi - \psi ^{\prime })(x_0) = \psi ^2(x_0) - {\psi '}^2(x_0) = (\psi (x_0) + \psi ^{\prime }(x_0))(\psi (x_0) - \psi ^{\prime }(x_0))= 0$ which implies that $\psi (y) = \psi ^{\prime }(y)$ whenever $\sigma y = x_0$. Proceeding inductively we deduce that $\psi (y) = \psi ^{\prime }(y)$ on the dense set of $y \in \cup _{n=1}^\infty \sigma ^{-n}x_0$, and this $\psi = \psi ^{\prime }$.

#### Remark 3.6

This simple argument doesn’t rule out the possibility of another non-positive fixed point.

### Analyticity

To show the analytic dependence of the solution we want to use the implicit function theorem applied to the function $G: \mathcal F_\theta \times \mathcal F_\theta \rightarrow \mathcal F_\theta$ defined by

$$\begin{aligned} G(\phi , \psi ) = \mathcal L_\phi \psi - \psi ^2. \end{aligned}$$

In order to apply the implicit function theorem at $(\phi _0, \psi _0) \in \mathcal F_\theta \times \mathcal F_\theta$ with $\psi _0>0$, say, satisfying $G(\phi _0, \psi _0)=0$ we need to show that $(D_2G)(\phi _0, \psi _0): \mathcal F_\theta \rightarrow \mathcal F_\theta$ is invertible. An easy calculation gives that

3.3 $$\begin{aligned} (D_2G)(\phi _0, \psi _0 ) = \left( \mathcal L_{\phi _0} - 2 \psi _0 \right) : \mathcal F_\theta \rightarrow \mathcal F_\theta . \end{aligned}$$

The spectral radius of any linear operator is the radius of the smallest disk (centred at the origin) containing the spectrum.

We recall the following result [9] which is also due to Ruelle.

#### Lemma 3.7

(Ruelle) The operator $\mathcal L_{\phi _0} : \mathcal F_\theta \rightarrow \mathcal F_\theta$ has spectral radius $\lambda _{\phi _0}$.

In particular, we see from Lemma 3.7 that $(\mathcal L_{\phi _0} - 2 \lambda _{\phi _0} {\mathbf 1})^{-1}: \mathcal F_\theta \rightarrow \mathcal F_\theta$ is a bounded linear operator since $2\lambda _{\phi _0}$ is not in the spectrum of $\mathcal L_{\phi _0}$. We can write

$$\begin{aligned} \begin{array}{ll} \left( \mathcal L_{\phi _0} - 2 \psi _0 \right) ^{-1} &{}= \left( (\mathcal L_{\phi _0} - 2 \lambda _{\phi _0}){\mathbf 1} + 2 (\lambda _{\phi _0}{\mathbf 1} -\psi _0) \right) ^{-1}\\ &{}= (\mathcal L_{\phi _0} - 2 \lambda _{\phi _0}{\mathbf 1})^{-1}\left( \sum _{n=0}^\infty \left( 2 (\lambda _{\phi _0} {\mathbf 1} -\psi _0)(\mathcal L_{\phi _0} - 2 \lambda _{\phi _0}{\mathbf 1})^{-1} \right) ^n\right) \end{array} \end{aligned}$$

which exists and is a bounded linear operator provided $\Vert \lambda _{\phi _0} {\mathbf 1} -\psi _0\Vert < \frac{1}{2\Vert (\mathcal L_{\phi _0} - 2 \lambda _{\phi _0} {\mathbf 1})^{-1} \Vert }.$

In particular, by (3.3) we see that $(D_2G)(\phi _0, \psi _0 )$ is invertible and thus the implicit function theorem applies. This allows us to deduce the analytic dependence.

#### Remark 3.8

(The Tangent Operator) Closely related to this circle of ideas is the use of a standard technique in understanding the iterates of a non-linear operator in a neighbourhood of a fixed point. More precisely, we consider the first order approximation to $G(\phi _0, \cdot ): \psi \mapsto \mathcal L_{\phi _0} \psi - \psi ^2$ where $\psi = \psi _0 + \epsilon \psi ^{(1)} + o(\epsilon )$. A simple calculation gives that the tangent operator

$$\begin{aligned} \mathcal T_{\phi _0} \psi := \lim _{\epsilon \searrow 0} \frac{G(\phi _0, \psi _0 + \epsilon \psi ^{(1)}) - G(\phi _0, \psi _0)}{\epsilon } = \mathcal L_{\phi _0} \psi ^{(1)} - 2\psi _0 \psi ^{(1)}. \end{aligned}$$

For definiteness, we can consider the specific case where we replace $\phi _0$ by $\phi _{1}$ as in Remark 1.7, then we see that the spectra $\hbox \mathrm{sp}(\mathcal T_{\phi _1})$ and $\hbox \mathrm{sp}(\mathcal L_{\phi _1})$ are simply related by $\hbox \mathrm{sp}(\mathcal T_{\phi _1}) = \hbox \mathrm{sp}(\mathcal L_{\phi _1}) -2$. Thus, since $\lambda _{\phi _1}=1$, by Lemma 3.7 the tangent operator $\mathcal T_{\phi _1}$ will have its spectra in the disk centred at $-2$ and of radius 1 (and thus outside the unit disk, except for the value $-1$). This suggests that the fixed point $\psi _{\phi _1}$ is locally unstable in a codimension one space under the iteration $G(\phi _1, \cdot )^n$

## Measures

The classical transfer operator $\mathcal L_\phi$ plays an important role in the ergodic theory of equilibrium states associated to $\phi$. More precisely, the equilibrium state is a fixed point for the dual $\mathcal L_{\phi _1}^*$ to the transfer operator $\mathcal L_{\phi _1}$ (satisfying $\mathcal L_{\phi _1}{\mathbf 1} = {\mathbf 1}$) for the associated function $\phi _1$ (cf. Remark 1.6). Although there is no direct analogue of equilibrium states in the context of the nonlinear equations $\mathcal L_\phi \psi = \psi ^2$ we have been studying, one can still use this identity to associate to the two functions $\phi$ and $\psi$ a natural invariant measure.

Given a solution $\mathcal L_\phi \psi = \psi ^2$ as in Theorem 1.5, we can consider the linear operator $\mathcal M_\phi : \mathcal F_\theta \rightarrow \mathcal F_\theta$ given by

$$\begin{aligned} (\mathcal M_\phi w)(x) = \sum _{\sigma y = x} e^{\phi (y)} \left( \frac{\psi }{\psi ^2\circ \sigma }\right) (y) w(y) \end{aligned}$$

which then satisfies $\mathcal M_\phi \mathbf 1 =\mathbf 1$ (cf. Remark 1.7). Since $\mathcal M_\phi$ is a transfer operator with a Hölder continuous potential, it is a consequence of the simplicity of the maximal positive eigenvalue for the operator in Theorem 1.3, and thus of its dual, that there is a unique $\sigma$-invariant probability measure $\mu$ such that $\mathcal M_\phi ^*\mu = \mu$, i.e., $\int f d\mu = \int \mathcal M_\psi f d\mu$ for all $f \in C^0(\Sigma )$. This leads to a non-standard version of the variational principle.

### Lemma 4.1

(Variational Principle for $\phi$ and $\psi$) For any $\sigma$-invariant probability measure $\nu$ we have that

4.1 $$\begin{aligned} \begin{array}{ll} h(\nu ) + \int \phi d\nu - \int \log \psi d\nu&\le 0 \end{array} \end{aligned}$$

with equality if and only if $\nu = \mu$.

### Proof

Let $\phi _2 : = \phi + \log \psi - 2\log \psi \circ \sigma$ then since $\mathcal M_\phi = \mathcal L_{\phi _2}$ satisfies $\mathcal L_{\phi _2} \mathbf 1 = \mathbf 1$ we see that $P(\phi _2)=0$ and $\mu$ is the unique equilibrium state associated to $\phi _2$, by Proposition 3.4 in [9]. Thus by the variational principle (Theorem 3.5 in [9]) we have

4.2 $$\begin{aligned} \begin{array}{ll} &{} h(\nu ) + \int \left( \phi + \log \psi - 2 (\log \psi )\circ \sigma \right) d\nu \\ &{}\quad = h(\nu ) + \int \phi _2 d\nu \\ &{} \quad \le P(\phi _2) = 0 = h(\mu ) + \int \phi _2 d\mu \\ &{} \quad = h(\mu ) + \int \left( \phi + \log \psi - 2 (\log \psi )\circ \sigma \right) d\mu \end{array} \end{aligned}$$

with equality if and only if $\mu = \nu$. By $\sigma$-invariance of the measures we have that $\int (\log \psi )\circ \sigma d\mu = \int \log \psi d\mu$ and $\int (\log \psi )\circ \sigma d\nu = \int \log \psi d\nu$ and thus (4.1) follows from (4.2). $\square$

We consequently have a particularly simple expression for the entropy $h(\mu )$.

### Corollary 4.2

We can write

$$\begin{aligned} h(\mu )= & {} \int (\log \psi ) d\mu - \int \phi d\mu . \end{aligned}$$
